# Comparison of Outcomes of Colorectal Polypectomy Using Traditional Snare and Rotary Snare: A Prospective Randomized Controlled Trial

**DOI:** 10.1155/2019/9123521

**Published:** 2019-10-17

**Authors:** Lu Xu, Zhixing Zhang, Jiarong Xie, Lei Xu, Weihong Wang

**Affiliations:** Department of Gastroenterology, Ningbo No. 1 Hospital, Ningbo 315010, China

## Abstract

**Background and Objective:**

The aim of the current study was to compare the efficacy and safety of polypectomy by using rotary snare *vs.* traditional snare during colonoscopy.

**Methods:**

A single-center randomized controlled trial, which included consecutive participants who were ≥18 years old and detected with polyp(s) during routine colonoscopy between July and September 2018, was conducted. Participants with colorectal polyps were randomized to receive polypectomy using rotary snares or traditional snares. The primary outcome measure was the comparison of the average time of removing a polyp between those two groups. The secondary outcome measure was to compare the polyp resection time by using SMSA (size, morphology, site, and access) scores.

**Results:**

A total of two hundred participants were included in this study. Of them, 100 participants were randomly assigned to the rotary snare group (214 polyps) and the other 100 participants were randomly assigned to the traditional group (232 polyps). The mean resection time was significantly shorter in the rotary snare group than in the traditional snare group (24.41 ± 18.14 seconds vs. 29.53 ± 25.74 seconds, *P* = 0.021). In the subgroup analysis, the resection time was also shorter in the rotary snare group than in the traditional snare group in SMSA level 1 (18.51 ± 8.26 seconds *vs.*23.84 ± 15.07 seconds, *P* = 0.013) and in SMSA level 2 (25.03 ± 15.32 seconds *vs.*29.15 ± 24.82 seconds, *P* = 0.009), respectively.

**Conclusion:**

Colorectal polyps could be removed more efficient by using rotary snares than by using traditional snares in SMSA level 1 and SMSA level 2.

## 1. Introduction

Colorectal cancer (CRC) is the third most commonly diagnosed cancer and the fourth leading cause of cancer death across the world [1]. In China, there are more than 250,000 cases of CRC newly diagnosed and nearly 140,000 CRC-related deaths every year [2]. Adenoma is often the precancerous lesion that progresses to the development of CRC. Colonoscopic polypectomy is the standard strategy for the interruption of the adenoma-to-carcinoma sequence worldwide [3, 4].

Previous studies showed that the colonoscopic removal of adenomatous polyps could significantly decrease the incidence and mortality of CRC [5–8]. At present, several techniques are available for polypectomy, such as hot or cold snare and hot or cold biopsy forceps [9]. Previous studies revealed that polyp resection used cold forceps, thus having a low risk of perforation, due to electrocautery was not be used, but it also led to lower removing efficacy (50%-80% complete removal rate of cases) [10]. Hot snare techniques have been reported to have a higher complete resection rate (over 95%), but with increased bleeding risk [11–13]. Hence, there is a lack of newer equipment for quick and safe polypectomy.

In China, hot snare polypectomy (HSP) and hot snare endoscopic mucosal resection (HS-EMR) with electrocautery are the most widely performed endoscopic procedures to remove colorectal polyps [14–17]. Given the large number of participants with polyps across China, newer polypectomy strategies with higher efficacy and lower complications are required. At present, a new snare technique, the rotary snare, has been developed for polypectomy. However, few clinical studies have compared the outcomes of rotary snare and traditional snare for polypectomy. Therefore, the aim of the current study was to compare the mean resection time for each polyp removal between rotary snares and traditional polypectomy snares. In addition, the study also assessed the safety of polyp resection using the rotary snare device.

## 2. Methods

### 2.1. Study Population and Design

The present study was a single-center, randomized controlled trial that comprised of participants detected with polyp(s) on routine colonoscopy between July and September 2018 in the Endoscopy Department of Ningbo First Hospital, Ningbo, China. Inclusion criteria in this study were as follows: (1) participants aged between 18 and 85 years and had colorectal polyp(s) on colonoscopy and (2) participants who provided a written informed consent. And the exclusion criteria were as follows: (1) participants had antiplatelet or anticoagulant therapy during the past one week of the procedure, (2) had coagulopathy, (3) had inflammatory bowel disease history, (4) with hemodynamic instability, and (5) pregnant participants. The enrolled participants were randomly assigned to the rotary polypectomy snare group or traditional polypectomy snare group. The random numbers were generated by computer using Excel software. Afterwards, researchers inserted the generated numbers into the sequential numbered envelopes and sealed them one by one. Participants detected with polyps during the colonoscopy examination were enrolled in the trial. Finally, the sealed envelope for each participant was opened, and participants were assigned either to the rotary snare group or traditional snare group according to the number. All participants provided a written informed consent. This study was in accordance with the Declaration of Helsinki principles [18] and CONSORT guidelines [19] and was approved by the institutional review board (2018-R009). This trial was registered at clinicaltrials.gov as NCT03608930.

### 2.2. Colonoscopy Procedures

Participants received 3 L polyethylene glycol (PEG) at five hours before the scheduled examination. A survey was conducted on all participants before the colonoscopy, including the following variables: age, gender, weight, height, and indication for colonoscopy [20]. During the colonoscopy procedure, the researcher evaluated the bowel preparation in accordance with the Ottawa Bowel Preparation Scale (OBPS) [21].

The endoscopist used a standardized colonoscopy procedure. All colonoscopies were performed using an Olympus PCF 290 video colonoscope (Olympus Inc., Japan) by one of the two experienced endoscopists, who have performed more than 1,500 colonoscopies. The complete cecal intubation was defined when the ileocecal valve and appendicular orifice were identified. Once colorectal polyps were confirmed during screening, the sealed envelope would be opened, and the participant was assigned to the corresponding group according to the number in the envelope. After cecal intubation, all the detected polyps during withdrawal were photographed. The location, size, and macroscopic type of all of polyps were documented according to the Paris classification [22]. The endoscopist estimated the size of the polyp using the snare diameter before each polypectomy.

In both groups, no analgesia or sedation was used during the procedure. In the traditional snare group, polypectomy was performed using hot snares with electrocautery. By using white light and narrow band imaging (NBI), the endoscopist estimated the selection of resections for polyp lesions as follows: (a) HS-EMR for sessile and flat polyp with 0-Is, IIa, or IIb morphology and (b) HSP for pedunculated polyps. For the HS-EMR, a normal saline solution was injected into the submucosal space to expand this layer, separating the mucosal lesion from the underlying muscularis propria by using a 25-gauge needle. Polypectomy was performed using hot snares with electrocautery. An ERBE 200D (Amco, Elektromedizin GmbH, Tübingen, Germany) was used in the ENDO CUT Q mode with the effect 4, length 1, interval 6, and forced coagulation current set at output limit 40 W and effect 2 for polypectomy. In the rotary polypectomy snare group (JHY-SD-23-230-30-A1; Wujin Economic Development Zone, Changzhou, China), the operating procedures were as follows: (1) intubation of the snare into the colonoscopy, (2) connection of the snare to the high-frequency device, (3) advancement of the sliding handle to open the loop, (4) advancement of the snare until it reached the target polyp and aligned by rotating the handle with the target polyp, (6) encirclement of the target polyp with the loop, (7) pulling of the sliding handle to lasso the target polyp, and (8) resection of the polyp by electrocautery, followed by catching of the transected polyp into a trap for histological evaluation. In the traditional polypectomy snare group (99052012232TW; MTM Endoskopie W. Haag KG, Goldsbergstrasse 18.46487 Wesel, Germany), except for the fourth step (angulation of the colonoscopy as required, advancement of the snare until the loop reached the target polyp), the rest of the process was the same as described for the rotary group. During the polypectomy, the research assistant used a stopwatch to record the resection time of each polyp (the stopwatch was started when the snare stretched out of the tube, and the stopwatch was ended when the polyp was completely removed). The margins of the polyps were carefully inspected with white light and narrow band imaging (NBI) in order to evaluate the completeness of the removal; any residual island of neoplasia was removed by snare. Only large polyps or suspected precancerous polyp-resected materials were retrieved for histologic examination in separate jars after the polypectomy. Furthermore, the time required for submucosal injection, electrocoagulation, and the use of titanium clips were excluded. Given that the number of polyps in each patient were different, the overall removing time of each participant would be different as well. Therefore, the overall polypectomy time in our study was only equal to the sum of the time of intubation and withdraw. In both groups, hemostatic clips were used to control the bleeding from the wound and in the hot snare polypectomy after removing the flat polyp.

### 2.3. Outcome Variables

The primary objective was to compare the mean resection time for one polyp between the two groups. The secondary outcome was to compare the mean resection time for one polyp according to the SMSA (size, morphology, site, and access) score [23]. Procedure-related complications, such as immediate bleeding (prolonged postpolypectomy bleeding > 30 seconds), delayed bleeding (any significant gastrointestinal bleeding requiring hospital admission or repeat endoscopy within two weeks), utilization rate of the hemoclip, perforation, and the need for additional endoscopic therapy, were also compared.

### 2.4. Sample Size Calculation

According to the recommendation of specialists, the proportion of participants that could finish the procedure in each group was assumed to be 85%, with anon-inferior margin of -15%. Therefore, it was estimated that 100 participants in each arm would be required based on a 95% 2-sided significance level, 80% power, and a 10% dropout rate.

### 2.5. Statistical Analysis

Continuous data was presented as mean ± SD, and categorical variables were presented as absolute and relative frequencies (percentage). Comparisons of demographic and clinical variables between males and females were performed using *t*-tests and *χ*^2^ tests, as appropriate. Statistical significance was defined as a *P* value of <0.05. All analyses were performed using SAS version 9.4 (SAS Institute, Cary, NC, USA).

## 3. Results

### 3.1. Baseline Characteristics

**Figure 1:**
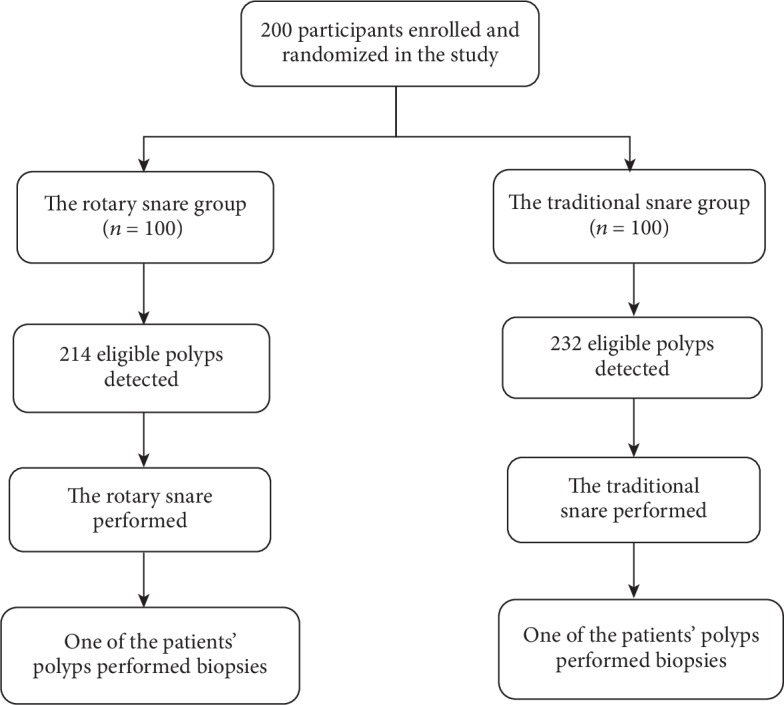
The flow chart of the whole process of this study.

Figure 1 presents the flow chart of the study. A total of 200 consecutive participants who had 446 colorectal polyps were enrolled. The participant characteristics and colonoscopy findings are presented in Table 1. No significant differences were observed in the baseline characteristics of participants between the two groups (age, gender, BMI, indication, and the quality of bowel preparation).

### 3.2. Polyp Characteristics

The mean number of polyps for each participant and the mean size of polyps were 2.23 mm and 7.22 ± 4.48 mm, respectively. Most of the resected polyps were tubular adenomas (69.5%). The characteristics of polyps are shown in Table 2. No significant statistical differences were observed between the two groups for the size, morphology, anatomical location, accessibility, and pathologic diagnosis.

### 3.3. Comparison of Primary Outcomes

**Figure 2:**
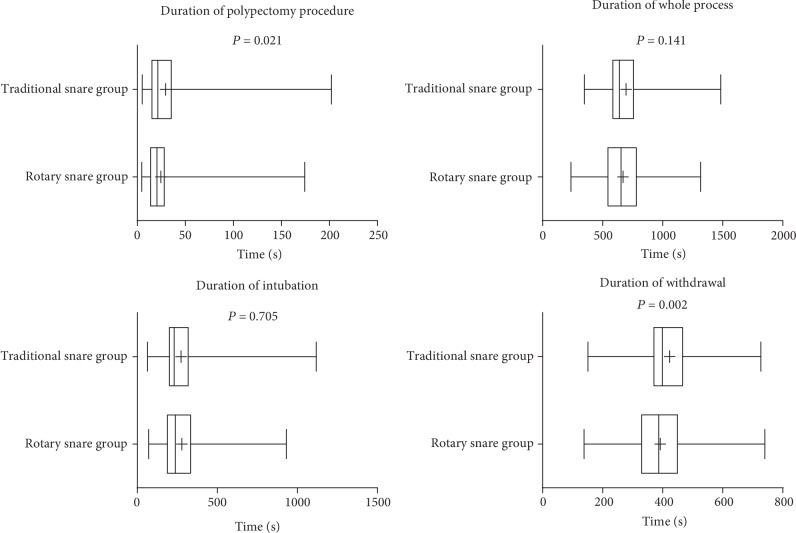
Comparison of each procedure time between the traditional snare group and rotary snare group.

The mean resection time for a single polyp was significantly shorter in the rotary snare group, when compared to the traditional snare group (24.41 ± 18.14 seconds *vs.*29.53 ± 25.74 seconds, *P* = 0.021; Figure 2). The time of withdrawal was 391.68 ± 119.35 seconds in the rotary snare group and 423.13 ± 100.42 seconds in the traditional snare group. There were no significant differences between the two groups with regard to the time required for complete colonoscopy and for intubation.

### 3.4. Comparison of Secondary Outcomes

**Figure 3:**
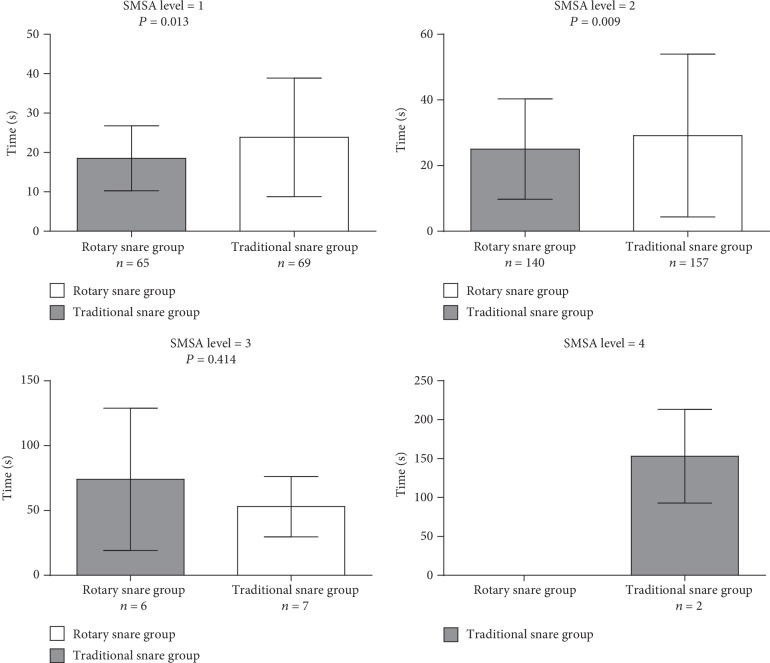
Comparison of polypectomy procedure times in size, morphology, site, and access (SMSA) score between the traditional snare group and rotary snare group.

Figure 3 present the single polyp resection time in different levels of difficulty as judged by the SMSA scoring system. The mean resection time was shorter in the rotary snare group, when compared to the traditional snare group for SMSA level 1 (18.51 ± 8.26 seconds *vs.*23.84 ± 15.07 seconds, *P* = 0.013) and SMSA level 2 (25.03 ± 15.32 seconds *vs.*29.15 ± 24.82 seconds, *P* = 0.009). However, no significant statistical differences were observed between SMSA levels 3 and 4.

### 3.5. Postpolypectomy Adverse Events

Table 3 showed the rate of immediate postpolypectomy bleeding in two groups, and the results showed no significant difference between the two groups. There were four cases of delayed postpolypectomy bleeding in the traditional snare group, but there were no cases in the rotary snare group. The endoscopic therapy and use of hemoclips for bleeding were similar in both groups. However, there were no perforations in either of the groups.

## 4. Discussion

This prospective randomized controlled trial demonstrated that using the rotary snare for colorectal polyps was technically feasible and has more favorable flexibility and shorter resection time, comparing to the traditional snares. For rotary snares, the direction of the snare could be adjusted by rotating the handle. Therefore, it is easier to reach and loop the target polyp, comparing to traditional snares. Furthermore, comparing with the traditional snare group, rotary snare improved the efficiency of the polypectomy, made the procedure theoretically safer, and significantly statistically shorten the mean polyp resection time for a single polyp. In the subgroup analysis, the resection time was also shorter in the rotary snare group in SMSA level 1 and SMSA level 2.

Snare polypectomy was found to be the preferred method for polypectomy in a survey of common gastroenterology practices [24]. A snare could be used either hot supplementing with electrocautery or cold without electrocautery. Previous studies assessed the completeness of an endoscopic resection and safety of cold snare polypectomy (CSP) through the use of different types of snare wires [25–27]. They found that CSP had the advantage of lesser complications and lower cost than HSP. However, HSP remains widely being used in clinical practice. Given that large numbers of colonoscopy and polypectomy screening in China, it is important to improve the efficiency of polypectomy and reduce the procedure time of the colonoscopy [14–17]. In the current study, the usage of rotary snares for participants undergoing HSP without sedation improved the efficiency of the polypectomy, and did not increase the adverse events, comparing to the traditional snares.

This study demonstrated that the mean resection time for a single polyp was shorter in the rotary snare group than in the traditional snare group. The possible reason was the difference between working principle of traditional snares and rotary snares. In cases of larger polyps or polyps located in remote areas of the colon, it is difficult to rotate the traditional snare and grasp polyps, and it is necessary for the endoscopist to adjust the position of the endoscope, which would prolong the operation time of the polypectomy. One of the advantages of rotary snare is that the loop can be rotated by the handle, which helps in looping the target polyp quickly, resecting the polyp with electrocautery, and then reducing the time for polypectomy.

Due to the relatively small sample size, the time for SMSA level 3 and SMSA level 4 difficult polyps had no statistical significance. Future larger sample size studies are needed to verify the feasibility of rotary snares for SMSA level 3 and SMSA level 4 difficult polyps. Besides, the potential advantage of the rotary snare is that the direction of the snare may be more accurate than the traditional snare, which could reduce the risk of complications, such as postoperative perforation and bleeding. However, no statistical difference in complications of the polypectomy between these two snares in the present study.

In previous study, immediate bleeding following polyp resection was not regarded as an adverse event only when it resulted in hospitalization, transfusion, or surgery [28–30]. While in order to assess the safety of polyp resection using the rotary snare device, we counted immediate bleeding as adverse events in the current study. However, the results showed a higher immediate bleeding rate in both traditional snare group and rotary snare group in our study, comparing to prior studies [13, 14, 16]. Mostly, this could be due to the intestinal mucosal oozing, which means oozing of blood after washing the site of resection, was considered equivalent to immediate bleeding in our study. Besides, in the traditional snare group, four participants had delayed bleeding, which was probably due to the consumption of improper diet, such as eating hard, craggy, or rough food after the polypectomy. All of them underwent emergency endoscopic treatment to successfully control the bleeding. Several risk factors, such as the recent usage of anticoagulants and hypertension, were reported to be associated with delayed bleeding in previous studies [31–33]. However, in the current study, those four cases did not have anticoagulants and hypertension at baseline. Therefore, the use of hard and craggy food after endoscopy could be the main cause of the delayed bleeding in our study.

Several limitations should be acknowledged. First, the study could not be blinded, because the endoscopist knew the usage of snare's type. Some biases were existed due to the different techniques used or other preexisting bias of the investigators. Second, the average polyp size in the present study was 7.22 ± 4.48 mm, while in the latest guidelines and studies, the polyp diameter < 10 mm should be resected by using CSP, in order to reduce the postoperative bleeding rate. Therefore, future studies are required to explore the role of rotary snares in CSP [34]. Third, the use of hemoclips in our study became a confounding factor in comparing the safety of these two types of snares. In addition, the study used EMR to remove the sessile or flat polyp to reduce the risk of bleeding and perforation. Since the overall polyp removal time was not calculated, it is not clear whether the rotary snare could reduce the overall operating time.

In conclusion, our study provided evidence that the efficacy and safety of rotary snares for the resection of colorectal polyps were better than the traditional snares. The mean resection time for a single polyp was shorter in the rotary snare group than in the traditional snare group, in SMSA level 1 and SMSA level 2. Therefore, the rotary polypectomy snare could be considered as a better device for colorectal polypectomy in clinical practice.

## Figures and Tables

**Table 1:** The characteristics of the included participants.

Characteristics	Overall	Snare type		*P* value
		The rotary snare	The traditional snare	
Age (years), mean ± SD	57.71 ± 11.95	56.89 ± 11.87	58.52 ± 12.02	0.325
Males, *n* (%)	130 (65.0)	61 (65.0)	69 (69.0)	0.562
Height (cm)	1.66 ± 0.07	1.65 ± 0.07	1.67 ± 0.08	0.464
Weight (kg)	64.50 ± 9.84	64.74 ± 9.25	64.26 ± 10.44	0.734
BMI (kg/m^2^), mean ± SD	23.26 ± 2.74	23.50 ± 2.82	23.02 ± 2.66	0.204
Indication, *n* (%)				0.538
Abdominal pain/discomfort	41 (20.5)	28 (28.0)	13 (13.0)	
Constipation	16 (8.0)	8 (8.0)	8 (8.0)	
Chronic diarrhea	52 (26.0)	25 (25.0)	27 (27.0)	
GI bleeding	12 (6.0)	5 (5.0)	7 (7.0)	
Physical examination	40 (20.0)	23 (23.0)	17 (17.0)	
Polyp review	35 (17.5)	10 (10.0)	25 (25.0)	
Other	4 (2.0)	1 (1.0)	3 (3.0)	
OBPS score, *n* (%)				0.314
≤4	126 (63.0)	78 (78.0)	48 (48.0)	
>5	74 (37.0)	22 (22.0)	52 (52.0)	

OBPS: Ottawa Bowel Preparation Scale.

**Table 2:** The characteristics of evaluated polyps.

Variables	Overall	Snare type		*P* value
		The rotary snare	The traditional snare	
No. of polyps evaluated	446	214	232	0.256
Polyps per patient, mean ± SD	2.23	2.14	2.32	0.685
Polyp size, mean ± SD	7.22 ± 4.48	6.77 ± 2.67	7.66 ± 5.74	0.178
<10 mm	365	171	194	
10-19 mm	71	36	35	
20-29 mm	9	4	5	
>30 mm	3	0	3	
Morphology, *n* (%)				0.916
Pedunculated	90 (20.2)	41 (19.4)	49 (20.9)	
Sessile	195 (43.7)	94 (44.5)	101 (43.0)	
Flat	161 (36.1)	76 (36.1)	85 (3 6.1)	
Anatomical location, *n* (%)				0.139
Right colon	195 (43.7)	102 (48.3)	93 (39.6)	
Left colon	251 (56.3)	109 (51.7)	142 (60.4)	
Access				0.577
Easy	96	43	53	
Difficult	350	168	182	
Pathologic diagnosis, *n* (%)				0.416
Tubular adenoma	139 (69.5)	70 (70.0)	69 (69.0)	
Tubulovillous adenoma	15 (7.5)	7 (7.0)	8 (8.0)	
Serrated adenoma	3 (1.5)	3 (3.0)	0 (0.0)	
Hyperplastic polyps	11 (5.5)	7 (7.0)	4 (4.0)	
Inflammatory polyp	32 (16.0)	13 (13.0)	19 (19.0)	

Right colon refers to proximal to splenic flexure; left colon refers to distal to splenic flexure.

**Table 3:** Adverse events per polypectomy.

Variables	Overall	Snare type		*P* value
		The rotary snare	The traditional snare	
Immediate bleeding	106	52	54	0.679
Endoscopic therapy	114	64	50	0.050
Hemoclip	361	174	187	0.938
Delayed bleeding	4	0	4	0.057
Perforation	0	0	0	—

Immediate bleeding defined as prolonged postpolypectomy bleeding (>30 seconds); delayed bleeding defined as any significant gastrointestinal bleeding requiring hospital admission or repeat endoscopy within 2 weeks.

## Data Availability

The original data in this study used to support the findings of this study are available from the corresponding author upon request.

## Supplementary Materials

Supplementary 1 Supplementary Figure 1: the working mechanism of the rotary snare. Supplementary Figure 2: the working mechanism of the traditional snare.

Supplementary 2 Supplementary video: the procedure of rotary snare polypectomy.
