# Supplementary figures and images for: Comparison of Outcomes of Colorectal Polypectomy Using Traditional Snare and Rotary Snare: A Prospective Randomized Controlled Trial
Source: Gastroenterol Res Pract. 2019 Oct 17;2019:9123521. doi: 10.1155/2019/9123521 (PMC6854937; doi:10.1155/2019/9123521)

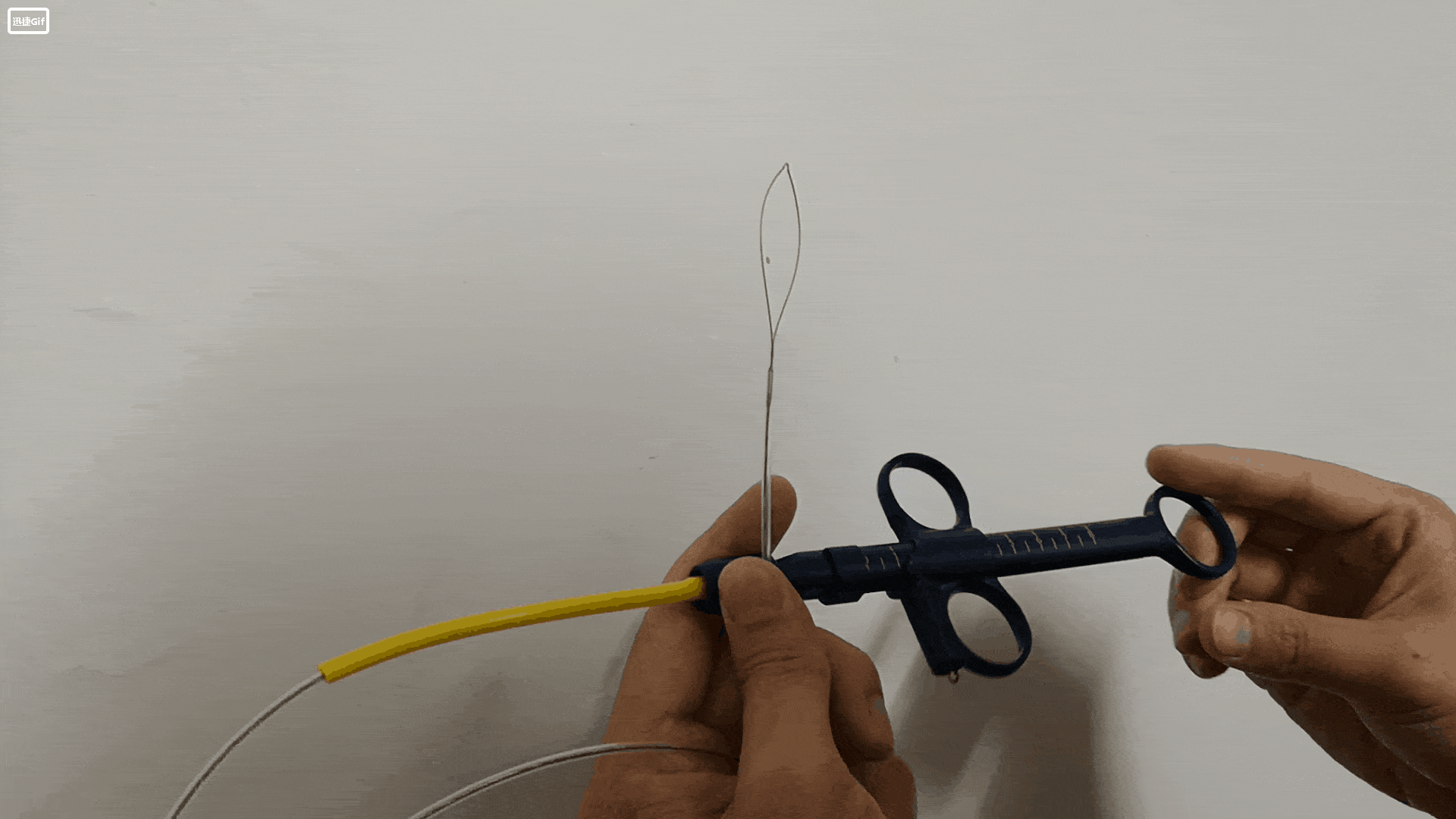

Supplement: Supplementary 1 — Supplementary Figure 1: the working mechanism of the rotary snare. Supplementary Figure 2: the working mechanism of the traditional snare. [file 9123521.f1.zip › Supplementary Figure 1. The working mechanism of the rotary snare-min.gif]

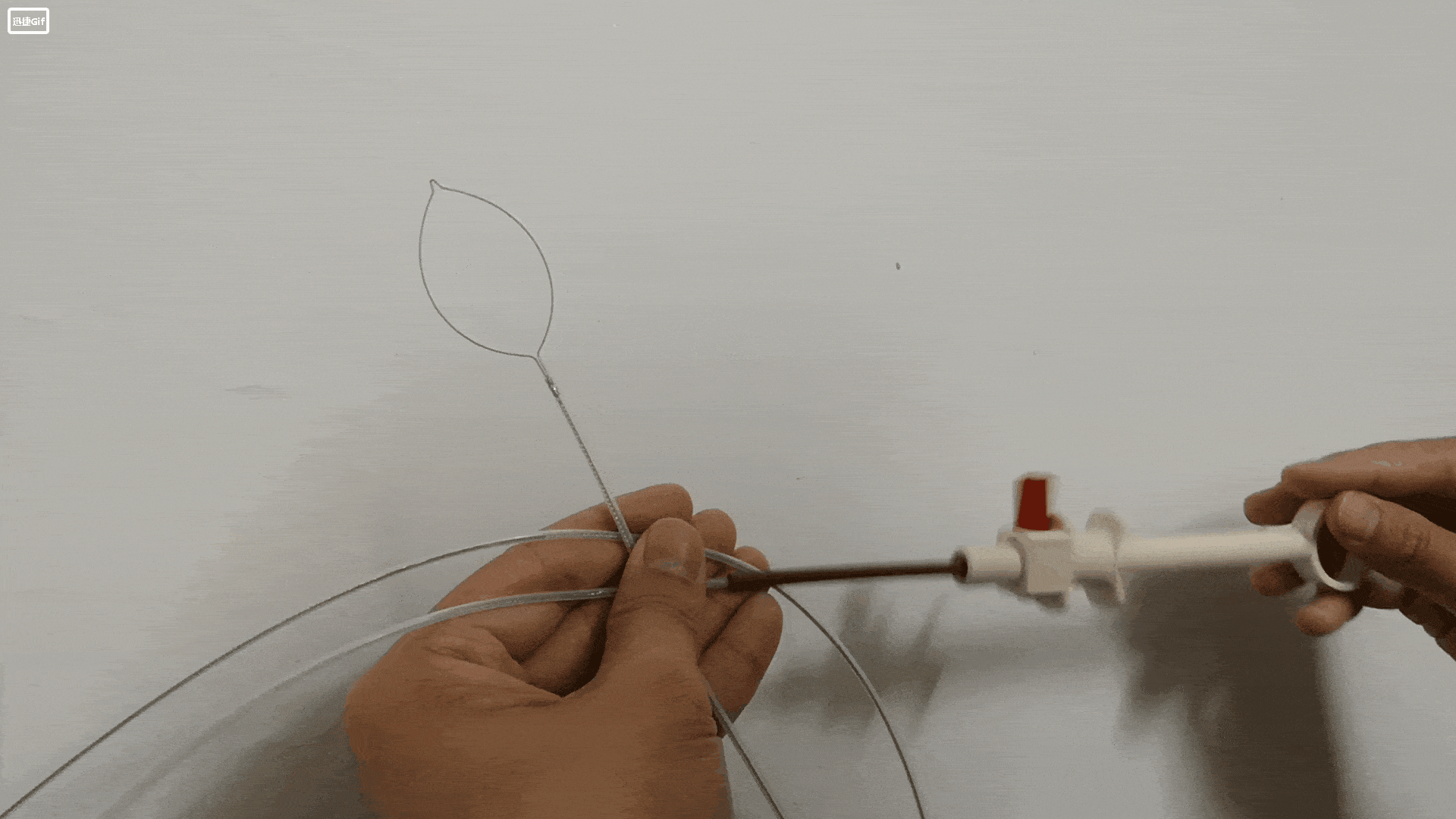

Supplement: Supplementary 1 — Supplementary Figure 1: the working mechanism of the rotary snare. Supplementary Figure 2: the working mechanism of the traditional snare. [file 9123521.f1.zip › Supplementary Figure 2. The working mechanism of the traditional snare-min.gif]
